# Development of cookies from wheat-yellow/white maize composite blends and their physical and sensory evaluation

**DOI:** 10.1371/journal.pone.0326532

**Published:** 2025-06-18

**Authors:** Muhammad Adnan, Masood Sadiq Butt, Imran pasha, Muhammad Shahid

**Affiliations:** 1 Faculty of Food, Nutrition and Home Sciences, National Institute of Food Science and Technology, University of Agriculture, Faisalabad, Faisalabad, Punjab, Pakistan; 2 Faculty of Sciences, Department of Biochemistry, University of Agriculture, Faisalabad, Faisalabad, Punjab, Pakistan; State University of Bangladesh, BANGLADESH

## Abstract

Wheat is a staple food in many countries globally, but it alone cannot meet the dietary demands of the growing population. To address this, there is an increasingly critical need to integrate alternative cereals into diets, enhancing nutritional diversity and reducing reliance on imports. In present study, various composite blends of wheat and maize were developed by taking different proportions of maize flour (10, 20, 30 and 40%) into the wheat flour and analyzed for proximate composition, phytates, minerals and pasting properties. By using these blends, cookies were made and tested for their physical and sensory evaluation. The crude fat, fiber and ash contents were increased significantly as the amount of maize into composite blends increased but insignificant for moisture, protein and NFE contents while reduction in the phytates was observed. The mineral analysis of various flours showed an increase in the calcium, magnesium, potassium and sodium while decrease in zinc contents. The pasting behaviour of wheat maize composite blends showed a significant decrease in their viscosities as the quantity of maize flour increased. The physical/textural parameters revealed a reduction in diameter and thickness whereas increase in hardness as the amount of maize supplementation increased which had diminishing affect at higher concentrations. It was observed that addition of maize flour into the wheat flour in the development of cookies at higher concentration negatively affected the sensorial attributes.

## Introduction

Cereals are regarded as a consistent source of energy for human beings. Calories provided by these major cereals in evolving and established nations are 60% and 30%, correspondingly. Globally, wheat, maize and rice are staple cereals which are critically important for the daily survival of major population [1]. The eating pattern of these cereal grains differs extensively in various regions of the earth; wheat is a desired grain cereal in the Middle East, Central Asia, South Asia, Europe, South and North America. However, rice is chiefly consumed in Asia and maize (corn) is an important cereal crop in Central America, Mexico, Eastern and Southern Africa [2].

Wheat is the 3^rd^ leading cereal grain of the world and believed to be one of the topmost cereal crops for contributing to the dietary necessities of more than 4 billion individuals. Its cropping areas like India, China and Australia are now subjected to elevated temperatures during their farming season which instigated reduction in the yield [3]. About 80% of wheat grain is utilized to develop many native foodstuffs like chapatti/roti and its variants called naans, tandoori roti, poories and parathas. Chapatti (locally known as roti) is a flat and unleavened hotplate baked foodstuff. Almost 21% of the wheat is being utilized to produce numerous industrial and bakery products, for example cakes, bread, pizza, pastries, and biscuits [4].

Maize is regarded as an important cereal crop, having a bounty of nutrients required to meet the needs of humans. The diversity of maize is relevant for food security, not merely in nutritive values but also owing to the phytochemical contents [5]. The main varieties of maize are floury maize, dent maize, sweet corn, Japanese stripped maize, blue corn, flour corn, waxy maize, pod corn, flint maize, etc. [6]. The most employed maize varieties are the yellow and white. Mostly, it is eaten roasted or boiled and consumed as a breakfast cereal [7]. Towards the end of 21^st^ century, yield of the cereal crops will be decreased because of change in the climate, excluding the millet due to its capability to endure severe climatic conditions [8].

Currently, the production of wheat has been prominently disturbed by changes in global climate across the world along with growing inaccessibility of water reserves. Pakistan is positioned among topmost ten nations that are more susceptible to the change in climate [9]. During the financial season 2020−21, the imports of wheat were assessed at 983.3 million US$. If the population of Pakistan propagates at the same rate of 1.9% then it will be a net importer of wheat in the coming years [10,11]. The variations in the consumption pattern in most of the populous Asian economies like Pakistan, Bangladesh, Indonesia, and India have been experienced, if the population continues to grow rapidly for the succeeding decades, it will increase the demand of food tremendously. Inflationary burdens on food expenditures have a substantial effect on food accessibility. Subsidization programs are delivered by managements to decrease the food expenses artificially [12].

Defatted flour made from maize germ can be a nutritive constituent of diet. It is a functional constituent which is incorporated into various types of flour to develop bread or chapatis [13]. Additionally, certain cereal foods like pasta are famous among everyone as they show appropriate sensual features as well as easy and rapid to formulate [14]. Composite flours are the combination of flours to substitute the wheat flour or whole wheat flour entirely/moderately with the alternative grains, tubers, legumes, or roots [15]. Consequently, numerous evolving nations have promoted the instigation of programs to assess the practicability of natively accessible cereal crops as an alternate of wheat [16]. Main ingredient utilized in the development of bakery-based items such as bread, pizza, cakes, cookies, *etc.* as well as pasta products is the wheat flour [17]. Novel composite blends/flours are being launched by evolving food companies as well as health professionals/nutritionists to fight undernourishment. The foremost characteristic for the exploitation of these blends is to manufacture various bakery or pastry flours to accomplish the dietary requirements. They revealed a beneficial impact on taste and texture along with the overall acceptability of the final product [18].

Yellow maize has higher amount of carotenoids, e.g., beta carotene, lutein etc. which has many health benefits as compared to the white maize. Likewise, colored maize has higher concentration of phenolics as well as the antioxidant activity [19]. The hybrids of maize have better yield and quality proteins contrasted to the maize varieties [20]. Moreover, yellow maize has higher amounts of magnesium, potassium and sodium opposed to the white maize that has higher quantity of calcium contents [21]. Maize was utilized up to 100% in various bakery food formulation, although, approximately 40% maize incorporation did not much affected the physical properties and consumer acceptance. The higher amount of maize negatively affected the rheological, textural and sensorial evaluation due to the dilution of gluten and viscous starch of wheat, leading to poor dough and its baking performance [22–24]. The purpose of this current research was to decrease the wheat import in the country and to add other cereal like maize into our diet. Therefore white and yellow maize flours were incorporated into wheat flour for the development of composite blends. These blends were investigated for their proximate composition, minerals, phytates and pasting parameters and subsequent cookies for physical and sensorial characteristics.

## Materials and methods

### Materials

After the screening of various maize varieties and hybrids (yellow and white), suitable cultivars were selected, i.e., yellow maize variety (NARC-2) and hybrid (FH-1036), while white maize variety (Jalal) and hybrid (Babar) were obtained from various local research institutes, i.e., Maize and Millet Research Institute (MMRI), Yusafwala, Cereal Crop Research Institute (CCRI), Pirsabak, National Agricultural Research Center (NARC), Islamabad. While, all the required raw materials procured from local market of Faisalabad.

### Preparation of wheat maize composite blends

Firstly, maize grains were subjected to cleaning, and then milling was done through a milling machine having the sieve size of 0.6 mm and rotational speed of 62 RPM (China Chakki; Model No. 6FTF-28 Mini Flour Mill). Later, composite blends were formulated by exploiting the maize flour of white and yellow maize (varieties and hybrids) at the rate of 10, 20, 30 and 40% into the wheat flour as mentioned in Table 1.

**Table 1:** Treatment plan of wheat maize composite blends.

Treatment	Wheat flour (%)	Maize flour (%)
T_0_ (Control)	100	–
T_1_	90	10^a^
T_2_	80	20^a^
T_3_	70	30^a^
T_4_	60	40^a^
T_5_	90	10^b^
T_6_	80	20^b^
T_7_	70	30^b^
T_8_	60	40^b^
T_9_	90	10^c^
T_10_	80	20^c^
T_11_	70	30^c^
T_12_	60	40^c^
T_13_	90	10^d^
T_14_	80	20^d^
T_15_	70	30^d^
T_16_	60	40^d^

^a^Yellow variety (NARC-2); ^b^Yellow hybrid (FH-1036)

^c^White variety (Jalal); ^d^White hybrid (Babar)

### Proximate composition

Wheat-maize composite blends and wheat flour were examined for their proximate composition that includes crude proteins, fats, ash contents, crude fibers, moisture contents, and nitrogen free extract (NFE) following the procedures of AACC (2010) [25].

### Mineral profile

Mineral contents of flour samples were determined according to protocols of AACC (2010). Accordingly, iron, calcium, and magnesium. were measured through atomic absorption spectrophotometer (AAS), whereas some minerals like potassium and sodium were analyzed via flame photometer. Purposely, a 0.5 gram dried maize sample was digested by di-acid mixture comprising HCl and nitric acid in the ratio of 7:3, correspondingly along with constant heating till solution became colorless. The resultant diluted solution was used for minerals determination.

### Phytic acid

Phytic acid present in composite blends and wheat flour was accessed through spectrophotometer (Cecil, CE-7200) following the work of Reichwald and Hatzack. About 0.05 g of sample was added to a 1.5 mL tube and 1 mL of 1 M HCl was dissolved. It was incubated on a thermo-mixer at 100 °C for 45 minutes under 1000 rpm. After the extraction process, the trial was centrifuged at 13000 rpm for five minutes. Around 500 µL aliquot of supernatant was moved to fresh tubes and 2 mL water (deionized) was mixed. Further, 800 µL of ferric solution was combined with 400 µL diluted extract/standards and tube was incubated at 100 °C on a thermo-mixer (300 rpm) for forty-five minutes. The sample was chilled for fifteen minutes to let the iron- phytate precipitate develop and centrifuged (13000 rpm) at 0 °C for ten minutes. Later, 600 µL aliquot was moved to micro-cuvette and then complexing mixture (800 µL) was inserted and absorbance was taken at 540 nm [26].

### Rapid visco analyzer (RVA)

It is an advancement of amylograph that illustrates complete curve of samples when it is subjected to heating and cooling cycles. Pasting properties of the wheat-maize composite blends were investigated through rapid visco analyzer (Perten RVA 2800, PerkinElmer, Shelton, USA). The prepared blend was transferred to RVA apparatus and examined for its various parameters such as hot paste viscosity, peak viscosity, set-back viscosity, and breakdown viscosity [27].

### Products development

The cookies were developed by exploiting the wheat-maize composite blends and wheat flour according to methods no. 10–50.05 as stated by AACC (2010). Cookies samples were developed by using the wheat/composite flour employing the following constituents, i.e., wheat/composite flour (250 g), vegetable ghee (125 g), refined sugar (125 g), milk powder (6 g), common salt (1.5 g), sodium bicarbonate (1.5), and water (33–39 g), respectively. The vegetable and refined sugar were combined thoroughly for 3–5 min in automated mixer to make a cream, afterwards 45 g eggs were incorporated and mixed moderately. In the meantime, sodium bicarbonate (1.5 g) was combined with wheat/composite flour (250 g) separately and then both of mixtures were added accompanied by water. Various food grade additives were incorporated in the mixing process following the treatment. Dough was kneaded and sheeted trailed by shaping into round form. Subsequently, sheeted dough stayed in a refrigerator for approximately 30 min. Finally, cookies samples were baked at 170–180 °C for 25–30 min in a hot air oven. The cookies were cooled down and kept in sealed containers till further use. Hot air convection oven with proper air flow will be used for baking. Oven will be preheated thoroughly for 10–15 minutes and trays were placed at proper distance in order to improve air flow and even baking of cookies. Then, multiple temperature sensors were placed at different strategic points (center, top front, top rear, bottom front, bottom rear) in the oven to record and calculate temperature consistency, ensuring uniform baking environment for product development. The loaves will be place at optimal distance in the trays to maintain even heat and air flow in the oven during the baking process.

### Physical attributes of cookies

#### Diameter and thickness.

Cookies were examined for their diameter and thickness through standard vernier calipers following the method no. 10–54.01 (AACC, 2010).

#### Color.

The color of the cookies were analyzed through the Hunter Lab colorimeter (Labscan XE, CM-3500d, Konica Minolta Ltd. Tokyo, Japan). L referred to the brightness, a* revealed greenness/redness, whereas b* showed the blueness/yellowness [28].

#### Hardness/firmness.

Texture analysis was performed after 15–20 minutes of product development. Hardness/firmness of cookies were measured by using the texture analyzer (TA.XTplus, Stable Microsystems, Surrey, UK) [29].

### Sensory evaluation

The cookies were analyzed for their sensorial characteristics using the 9-point hedonic scale (from extremely dislike to extremely like). The parameters during the sensory were color, flavor, taste, texture, and overall acceptability [30]. A panel of 10 trained individuals (5 males and 5 females) having experience of cereal products evaluation performed the double blind testing of the cookies samples. The individuals for the sensory evaluation will be from 18–60 years of age, non smokers, no allergic to ingredients in cookies such as wheat, maize, eggs, etc. with 25–40 hours of sensorial training over 3–6 weeks in cereal based cookies. Sensory evaluation will be performed in temperature controlled (22–25°C), quiet, odourless, well lit environment having separate booths to ensure minimal interaction. Moreover, double blind environment will be employed to overcome the biasness.

The following 9–point hedonic scale will be used for sensory scoring;

(1–Dislike extremely, 2–Dislike very much, 3–Dislike moderately, 4–Dislike slightly, 5–Neither like/dislike, 6–Like slightly, 7–Like moderately, 8–Like very much, 9–Like extremely).

### Statistical analysis

Finally, all the collected outcomes were subjected to a statistical evaluation for the measuring the significance level using the completely randomized design (CRD). Furthermore, post-hoc means comparison was performed by Tukey’s test [31]. The software used for performing the statistical evaluation was statistix 8.1.

## Results and discussion

### Proximate composition of the blended flour

Analysis revealed highly significant variations in crude fat, fiber and ash contents, whereas moisture, crude protein and nitrogen-free extract (NFE) contents showed no statistically significant differences. The mean values of proximate composition of composite blends and wheat flour are displayed in Table 2. The concentration of crude protein varied from 11.86 to 13.07%, peak value was observed in T_0_ while lowermost in T_12_ comprising 40% maize flour of white variety (Jalal). Crude fat of composite blends revealed that maximum amount was shown by T_4_ followed by T_8_, T_12_ and T_16_. Furthermore, the findings of crude fiber exposed that highest concentration of fiber was present in T_8_ and T_12_ containing 40% maize flour. The ash contents of composite blends fluctuated from 0.65 to 1.15% that was observed highest in the T_4_. Similarly, among the NFE contents of wheat maize blends, peak value was noticed in T_0_ followed by T_13_, T_1_ and T_5_. The increase in crude fat, crude fiber and ash contents in composite flours was due to the higher amount of fat, fiber and ash contents in the maize flour as compared to the refined wheat flour [32].

**Table 2:** Proximate composition (%) of wheat and wheat maize composite blends.

Treatment	Moisture	Crude protein	Crude fat	Fiber	Ash	NFE
T_0_	12.69 ± 0.64	13.07 ± 0.43	1.24 ± 0.08^i^	0.93 ± 0.04^g^	0.65 ± 0.06^f^	74.38 ± 0.91
T_1_	12.39 ± 0.58	12.85 ± 0.67	1.43 ± 0.11^ghi^	1.02 ± 0.005^fg^	0.76 ± 0.04^def^	73.89 ± 1.20
T_2_	12.26 ± 0.29	12.62 ± 0.47	1.77 ± 0.08^def^	1.08 ± 0.06^cdefg^	0.90 ± 0.05^bcde^	72.96 ± 1.14
T_3_	12.01 ± 0.38	12.23 ± 0.71	2.06 ± 0.11^abc^	1.17 ± 0.07^bcdef^	1.08 ± 0.06^ab^	72.19 ± 0.79
T_4_	11.86 ± 0.62	11.88 ± 0.75	2.34 ± 0.07^a^	1.25 ± 0.06^abcde^	1.15 ± 0.07^a^	71.65 ± 1.43
T_5_	12.54 ± 0.51	12.89 ± 0.67	1.35 ± 0.03^i^	1.07 ± 0.04^defg^	0.74 ± 0.05^def^	73.75 ± 0.94
T_6_	12.35 ± 0.32	12.74 ± 0.56	1.65 ± 0.06^fgh^	1.21 ± 0.09^abcdef^	0.87 ± 0.06^cde^	73.11 ± 1.39
T_7_	12.20 ± 0.71	12.56 ± 0.49	1.99 ± 0.09^cde^	1.27 ± 0.05^abcd^	1.06 ± 0.04^ab^	72.64 ± 0.61
T_8_	12.10 ± 0.27	12.10 ± 0.60	2.29 ± 0.10^ab^	1.41 ± 0.10^a^	1.11 ± 0.08^a^	71.98 ± 1.78
T_9_	12.65 ± 0.49	12.80 ± 0.54	1.40 ± 0.04^hi^	1.04 ± 0.09^efg^	0.71 ± 0.03^ef^	73.71 ± 1.14
T_10_	12.55 ± 0.52	12.61 ± 0.83	1.73 ± 0.10^ef^	1.12 ± 0.10^cdefg^	0.89 ± 0.05^bcde^	73.14 ± 1.39
T_11_	12.45 ± 0.56	12.37 ± 0.40	2.02 ± 0.12^bcd^	1.24 ± 0.06^abcde^	1.01 ± 0.07^abc^	72.57 ± 1.55
T_12_	12.36 ± 0.91	11.86 ± 0.67	2.29 ± 0.08^ab^	1.38 ± 0.08^ab^	1.08 ± 0.06^ab^	72 ± 1.28
T_13_	12.54 ± 0.62	12.85 ± 0.71	1.38 ± 0.07^hi^	1.01 ± 0.07^fg^	0.75 ± 0.04^def^	73.9 ± 0.96
T_14_	12.28 ± 0.49	12.72 ± 0.65	1.69 ± 0.11^fg^	1.09 ± 0.05^cdefg^	0.91 ± 0.07^bcd^	73.42 ± 0.71
T_15_	12.1 ± 0.77	12.49 ± 0.38	2.04 ± 0.11^bcd^	1.19 ± 0.04^abcdef^	1.01 ± 0.07^abc^	72.94 ± 1.17
T_16_	11.98 ± 0.35	12.27 ± 0.77	2.27 ± 0.08^abc^	1.30 ± 0.08^abc^	1.10 ± 0.08^a^	72.27 ± 1.05
F Value	0.57^ns^	0.98^ns^	49.1^**^	10.4^**^	19.4^**^	1.3^ns^

Values are represented as Mean±SD. Means bearing different superscript varied significantly

(P<0.01) ^**^ = Highly Significant (P>0.05) ^ns^ = Non Significant

A study conducted on composite wheat maize flour porridge showed that there was a decrease in the crude protein contents with increasing the maize concentration. The maximum crude protein was detected in 100% wheat flour specified that it had a substantial quantity of protein as compared to maize. Moreover, crude fat contents were augmented by increasing the proportion of maize flour which fluctuated between 1.82 ± 0.04% and 2.48 ± 0.02%. Additionally, there is an increase in the crude fiber contents with increasing the maize due to the high amount of crude fiber in it [33]. Another research on the cereal blends comprising maize and wheat flour exposed that there was an increase in fat contents by enhancing the amount of yellow maize flour [34].

A comparable study on the composite flour having flour of corn, pumpkin and soybean revealed that fat content was increased with enhancing the amount of them. Moreover, ash and fiber contents were increased however carbohydrates were decreased significantly by replacing the wheat flour with corn, soybean and pumpkin flour [35]. A similar kind of investigation on wheat maize composite flour, in which they found that moisture contents were insignificant among the treatments. The crude fiber contents was fluctuated from1.44–8.75%, which was increased by increasing the amount of brewer’s spent grain flour (BSGF) and maize flour significantly. Similarly, crude protein was affected substantially (increased) by BSGF while maize flour did not contribute to crude protein considerably. The ash contents of composite flour were also increased significantly by enhancing the amount of BSGF and maize flour which was ranged from 0.69–2.50% [36].

### Minerals analysis

The mean square values of all the minerals exposed a highly significant fluctuation among various treatments of composite blends and wheat flour (Table 3). The mean values of calcium revealed that maximum amount of calcium was present in T_4_ followed by T_3_ and T_8_ whereas minimum concentration was noticed in T_0_ (13.06 ± 0.46 mg/100 g). Additionally, among the magnesium contents of the composite blends, greatest quantity was depicted by T_8_ and least value was found in T_12_ followed by T_11_ and T_10_. The iron contents of the treatments revealed that its amount was fluctuated from 1.74–2.51 mg/100 g which was found uppermost in the treatment T_4_ followed by T_8_, T_3_ and T_7_ while lowest concentration was detected in T_16_ succeeded by T_12_ and T_15_. Moreover, findings of zinc contents disclosed that the topmost amount of zinc was shown by T_0_ and T_5_ however least concentration was noticed in T_16_ and T_12_. The mean values of potassium content fluctuated from 115.93 to 189.56 mg/100 g which was highest in the T_8_ followed by T_4_ and bottommost value was shown by the control having 100 wheat flour. Additionally, T_0_ had the least amount of sodium (2.52 ± 0.10 mg/100 g) among the treatments of composite blends trailed by T_5_ and T_1_ and greatest concentration was found in T_16_ followed by T_12_ and T_15_.

**Table 3:** Mineral composition (mg/100 g) of wheat and wheat maize composite blends.

Treatment	Calcium	Magnesium	Iron	Zinc	Potassium	Sodium
**T**_**0**_	13.06 ± 0.46^de^	79.21 ± 1.15^defg^	2.19 ± 0.06^cdefg^	1.68 ± 0.06^a^	115.93 ± 2.61^g^	2.52 ± 0.10^j^
**T**_**1**_	13.79 ± 0.46^cde^	81.92 ± 1.72^bcdef^	2.24 ± 0.07^bcdef^	1.64 ± 0.08^ab^	126.58 ± 2.31^fg^	3.48 ± 0.11^hi^
**T**_**2**_	14.66 ± 0.38^abcde^	84.30 ± 2.52^abcd^	2.33 ± 0.04^abcd^	1.59 ± 0.09^abcd^	138.95 ± 4.24^de^	4.27 ± 0.12^fg^
**T**_**3**_	15.74 ± 0.78^ab^	86.70 ± 1.69^abc^	2.41 ± 0.0.09^abc^	1.49 ± 0.06^abcde^	162.95 ± 3.30^bc^	5.06 ± 0.22^cde^
**T**_**4**_	16.30 ± 0.52^a^	88.31 ± 2.88^ab^	2.51 ± 0.0.08^a^	1.38 ± 0.02^cde^	186.20 ± 4.78^a^	5.68 ± 0.26^b^
**T**_**5**_	13.47 ± 0.74^cde^	83.56 ± 1.57^abcde^	2.21 ± 0.06^bcdef^	1.67 ± 0.09^a^	128.62 ± 1.28^ef^	3.24 ± 0.14^i^
**T**_**6**_	13.92 ± 0.68^cde^	85.20 ± 2.67^abcd^	2.27 ± 0.07^abcde^	1.63 ± 0.06^ab^	141.90 ± 2.46^d^	4.08 ± 0.21f^g^
**T**_**7**_	14.70 ± 0.63^abcd^	87.34 ± 2.45^abc^	2.36 ± 0.05^abc^	1.49 ± 0.09^abcde^	164.62 ± 2.85^b^	4.99 ± 0.17^de^
**T**_**8**_	15.10 ± 0.72^abc^	90.15 ± 3.50^a^	2.44 ± 0.10^ab^	1.37 ± 0.03^de^	189.56 ± 4.16^a^	5.54 ± 0.19^bc^
**T**_**9**_	12.94 ± 0.40^e^	79.06 ± 1.78^defg^	2.11 ± 0.09^defgh^	1.61 ± 0.09^abc^	122.62 ± 3.29^fg^	3.80 ± 0.11^gh^
**T**_**10**_	13.36 ± 0.33^cde^	77.22 ± 2.40^efg^	2.01 ± 0.07^fghij^	1.50 ± 0.10^abcde^	132.34 ± 2.08^def^	4.54 ± 0.13^ef^
**T**_**11**_	13.97 ± 0.53^cde^	75.87 ± 1.41^fg^	1.93 ± 0.8^hijk^	1.42 ± 0.04^bcde^	153 ± 2.89^c^	5.36 ± 0.08^bcd^
**T**_**12**_	14.78 ± 0.84^abcd^	74.49 ± 1.73^g^	1.78 ± 0.03^jk^	1.30 ± 0.05^e^	168.13 ± 5.63^b^	6.43 ± 0.28^a^
**T**_**13**_	13.61 ± 0.22^cde^	80.81 ± 2.09^cdefg^	2.05 ± 0.13^efghi^	1.60 ± 0.10^abcd^	122.96 ± 1.50^fg^	4.18 ± 0.22^fg^
**T**_**14**_	13.88 ± 0.64^cde^	81.66 ± 2.44^bcdef^	1.96 ± 0.11^ghijk^	1.48 ± 0.09^abcde^	132.59 ± 2.43^def^	5.30 ± 0.11^bcd^
**T**_**15**_	14.35 ± 0.43^bcde^	83.34 ± 1.72^bcde^	1.85 ± 0.07^ijk^	1.39 ± 0.08^cde^	153.09 ± 4.18^c^	6.41 ± 0.18^a^
**T**_**16**_	14.44 ± 0.58^bcde^	84.75 ± 2.63^abcd^	1.74 ± 0.04^k^	1.28 ± 0.06^e^	165.55 ± 6.19^b^	6.80 ± 0.17^a^
**F value**	7.47**	11.9**	25.7**	8.32**	120**	135**

Values are represented as Mean±SD. Means bearing different superscript varied significantly

(P<0.01) ^**^ = Highly Significant

The increased levels of potassium, magnesium and calcium can be attributed to their higher amounts in the maize compared to wheat [32,37]. According to a study conducted on composite flour biscuits containing wheat, maize, coconut and almond flour, the amount of calcium was ranged from 3.68–8.66 mg/100 g, magnesium 2.42–5.70 mg/100 g, potassium 5.64–10.17 mg/100 g, iron 1.45–6.74 mg/100 g and zinc 3.27–7.85 mg/100 g, respectively. The surge in minerals in composite-flour biscuits might be owing to the greater amount of these minerals in maize, almond and coconut flour than the wheat flour [38].

### Phytic acid

**Fig 1:**
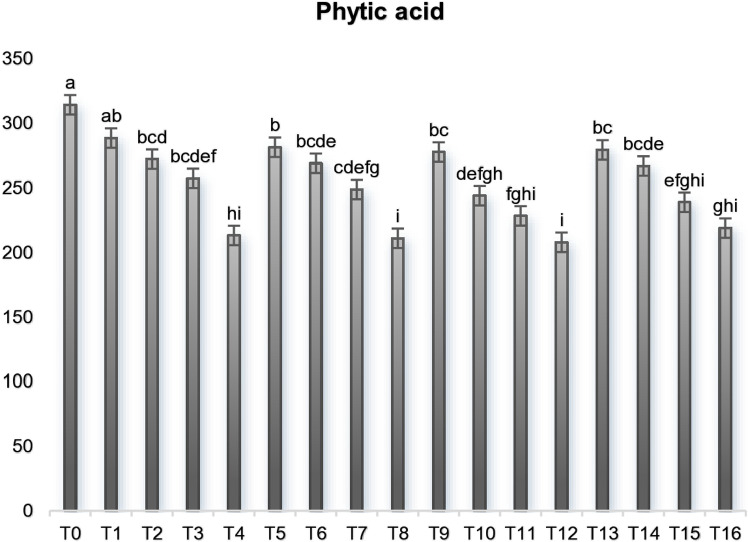
Phytic acid contents (mg/100 g) of control and wheat maize composite cookies. The data is presented as means. Means bearing different superscript varied significantly (P < 0.01) ^**^ = Highly Significant.

Phytic acid is a plentiful plant component which is present in edible legumes, cereals, nuts, and oil seeds. The amount of these antinutritional compounds depends upon various factors such as growing circumstances, harvesting methods, processing approaches, investigation procedures as well as stage of crop/food being examined. The phytate contents are condensed in aleurone layer of the grains mostly, making them more concerted in the bran portion [39]. Furthermore, research on positive health and biological effects recommends that these phytates might have advantageous influences on numerous other ailments, for instance vascular calcification inhibition and accordingly reduced the chances of cardiovascular diseases (CVDs), diabetes, coronary diseases, kidney stones treatment and Parkinson’s disease [40]. The outcomes of these phytates showed that they exhibited a very substantial variation among the treatments The mean values of phytic acid (Fig 1) revealed that wheat flour had the highest concentration of these compounds as found in T_0_ treatment (314.22 ± 10.27 mg/100 g) trailed by T_1_ (288.48 ± 13.65 mg/100 g), T_5_ (281.35 ± 12.77 mg/100 g), T_13_ (279.31 ± 12.94 mg/100 g) and T_9_ (277.65 ± 11.64 mg/100 g). The amount of phytic acid contents was decreased by elevating the concentration of maize flour into the wheat for developing the composite blends due to its higher amount in wheat [41,42].

An investigation performed by Yaver and Bilgicli (2019) on various types of flours along with the composite flour, wherein they found that maximum phytates contents were observed in soybean flour (2520 mg/100 g) while the lowermost amount was shown by the common flour/wheat flour (370 mg/100 g) [43]. A parallel trend of outcomes was observed in the research of Sparvoli et al. (2016) who used the common bean and maize flour into wheat flour for the development of biscuits [44]. Our findings were correlated with the study of Udomkun et al. during the assessment of phytates of composite flours. The phytate contents in control having 25 g wheat flour per 100 g composite flour (30 g maize flour, 25 g wheat flour, 15 g sorghum flour, 10 g sesame flour and 20 g soy flour) were 170.8 mg/100 g, while the highest concentration (211.6 mg/100 g) were detected in P-R composite blend containing 15 g wheat into 100 g rice flour and least amount (142.4 mg/100 g) was revealed by F-C composite blend comprising 25 g wheat flour per 100 cassava flour [45].

### Rapid visco analyzer

Mean and standard deviation of pasting properties such as peak viscosity, trough viscosity, *etc.* of various treatments were presented in Table 4. Peak viscosity is the capability to swell easily prior to their physical collapse and specifies the power of pastes developed in the gelatinization process. Trough viscosity is the lowest value in a continuous temperature of rapid visco-analyzer, analyzing the capacity to resist the breakdown during the cooling process. Breakdown viscosity is stated as the degree of disintegration of starches or their pastes stability in heating course. Setback viscosity is regarded as retrogradation tendency of the paste developed through a starchy type of food. These viscosities of composite blends revealed that maximum values were shown by control having only wheat flour, whereas lowermost values were detected in T_8_ containing 60% wheat flour and 40% maize flour of yellow maize hybrid (FH-1036). Consequently, it was concluded from the outcomes that by enhancing the amount of maize flour into the wheat has a diminishing effect on all type of viscosities.

**Table 4:** Pasting properties of wheat and wheat maize composite blends.

Treatment	Peak viscosity (cP)	Trough viscosity (cP)	Breakdown viscosity (cP)	Final viscosity (cP)	Setback viscosity (cP)
**T**_**0**_	3011.40 ± 116.08^a^	1965.77 ± 78.37^a^	1142.29 ± 56.81^a^	3540.30 ± 131.89^a^	1503.16 ± 68.81^a^
**T**_**1**_	2903.40 ± 59.88^ab^	1899.57 ± 65.92^ab^	1074.28 ± 49.21^ab^	3368.77 ± 111.05^ab^	1404.59 ± 72.53^ab^
**T**_**2**_	2754.35 ± 125.26^abc^	1832.07 ± 46.18^abcd^	908.73 ± 21.35 cd	3145.83 ± 90.59^bc^	1232.63 ± 42.71^cde^
**T**_**3**_	2534.61 ± 106.39^cde^	1763.23 ± 84.32^bcdef^	741.09 ± 42.57^e^	2873.07 ± 102.62^cde^	1039.70 ± 44.39^f^
**T**_**4**_	2198.83 ± 39.43^fg^	1640.47 ± 14.79^efg^	614.43 ± 26.1^efg^	2449.98 ± 113.98^fgh^	802.48 ± 29.53^g^
**T**_**5**_	2851.08 ± 127.54^ab^	1862.74 ± 36.81^abc^	1014.52 ± 66.92^bcd^	3246.3 ± 75.32^ab^	1341.94 ± 61.78^bcd^
**T**_**6**_	2642.45 ± 41.95^bcde^	1776.21 ± 45.34^bcdef^	888.21 ± 50.58^d^	3052.65 ± 124.68^bcd^	1176.94 ± 21.31^e^
**T**_**7**_	2399.10 ± 81.46^ef^	1657.97 ± 74.55^defg^	708.46 ± 14.21^ef^	2700.72 ± 132.09^efg^	960.25 ± 46.34^f^
**T**_**8**_	2071.94 ± 93.58^g^	1562.30 ± 61.69^g^	561.07 ± 23.42^g^	2350.10 ± 103.93^h^	748.90 ± 24.17^g^
**T**_**9**_	2878.44 ± 108.84^ab^	1878.01 ± 73.52^ab^	1027.71 ± 46.84^abc^	3305.32 ± 142.25^ab^	1356.07 ± 40.06^bc^
**T**_**10**_	2668.78 ± 75.40^bcde^	1794.77 ± 87.03^abcde^	896.56 ± 43.95^d^	3078.39 ± 110.5^bc^	1192.80 ± 16.31^e^
**T**_**11**_	2435.66 ± 115.93^def^	1682.68 ± 70^cdefg^	725.08 ± 35.96^ef^	2724.81 ± 89.55^def^	974.32 ± 55.12^f^
**T**_**12**_	2105.19 ± 49.34^g^	1593.68 ± 50.11^fg^	579.92 ± 37.49^g^	2358.16 ± 88.62^h^	781.08 ± 22.19^g^
**T**_**13**_	2889.97 ± 109.26^ab^	1891.26 ± 22.93^ab^	1050.77 ± 66.34^ab^	3335.95 ± 128.26^ab^	1382.25 ± 37.52^ab^
**T**_**14**_	2688.90 ± 95.34^bcd^	1825.88 ± 55^abcd^	900.62 ± 19.42^d^	3094.62 ± 74.45^bc^	1210.89 ± 56.15^de^
**T**_**15**_	2460.54 ± 60.45^def^	1748.67 ± 71.43^bcdef^	735.40 ± 33.44^e^	2742.76 ± 109.49^def^	1000.75 ± 41.40^f^
**T**_**16**_	2119.40 ± 74.68^g^	1627.13 ± 26.14^efg^	598.19 ± 19.69^fg^	2372.66 ± 79.34^gh^	796.67 ± 27.29^g^
**F value**	33.7**	11.7**	62.2**	38.6**	89.3**

Values are represented as Mean±SD. Means bearing different superscript varied significantly

(P<0.01) ^**^ = Highly Significant

The starch in maize is more crystalline and less gel forming compared to the starch of wheat that has higher amount of amylopectin. Therefore, with the increase in the maize contents, a reduction in the ability of the flour to thicken might be observed when heated Thus, increasing the maize into the wheat flour from 10% to 40% resulted in a decrease in gelatinization and pasting attributes (lower viscosity and weaker gel formation), as well as an alteration in texture (from elastic to crumbly) [46–48]. In the wheat maize composite blends, functional compounds of wheat such as gluten and high viscous starch are diluted with maize starch which caused the reduction in the peak viscosity, particularly more at higher levels (30–40%). Furthermore, high amylose and lower amylopectin in the maize starch bring down the viscosity development. This lower water retention in composite blends lowered the gelatinization process that decrease the final or peak viscosity. Similarly, starch in maize retrogrades quicker compared to the wheat, resulted in lesser final and setback viscosities [49].

Same pasting behavior was reported in the study of composite flour consisting rice and cornsilk flour. Incorporation of cornsilk into the rice flour significantly altered the pasting characteristics of that composite flour. The addition of cornsilk into wheat flour decreased the peak, trough, final, setback viscosities as well as the peak time [50]. A research conducted on the pasting behavior of composite flours comprising wheat flour and millet reported that peak viscosity of adjusted composite flours fluctuated from 358–462 RVU. The value of peak viscosity of the composite blends were increased by enhancing the concentration of pearl-millet contents into the wheat flour. Control (wheat flour 100%) revealed the very highest value of peak viscosity. The trough viscosity varied from 354–442 RVU in composite flour whereas control had 936 RVU. Similarly, final viscosity fluctuated from 782 to 940 RVU for composite blends, however its value for 100% wheat flour sample was 1906 RVU [51]. A similar trend of decreasing viscosities was reported during the study of multigrain bread. Control had the maximum values of viscosities as compared to the composite blends which had flour from non-wheat sources such as amaranth and acha grains [52].

### Physical attributes of cookies

The investigation of physical features of cookies exhibited a decidedly substantial difference among the treatments and their mean values were presented in Table 5. The maximum value of diameter was observed in control and least diameter was depicted in T_16_. The diameter was decreased by enhancing the amount of maize flour addition in the wheat flour. Likewise, thickness was reduced with the enhancement of maize flour replacement, highest level of thickness was noticed in T_0_ followed by T_1_, T_9,_ T_5_ and T_13_ whereas least value was noticed in T_8_. The decrement in thickness and diameter of wheat maize cookies might be due to the grainy texture and contrasting water absorption characteristics of maize flour, which prevent the spreading of dough. Therefore, the reduction in the thickness and growth in the diameter were largely owing to dilution effect on the gluten network. The resultant cookies tend to have denser, slightly thicker and smaller diameter as compared to common or wheat wheat cookies [53,54]. Parallel findings were reported in composite flour cookies having wheat and pearl millet flour, in which they observed that there was a reduction in thickness as well as the diameter of cookies [55].

**Table 5:** Physical characteristics of wheat and wheat maize composite cookies.

Treatment	Diameter (mm)	Thickness (mm)	Hardness (N)	Color L values	Redness (a*)	Yellowness (b*)
**T**_**0**_	42.46 ± 0.85^a^	5.39 ± 0.13^a^	12.26 ± 0.10^h^	57.73 ± 0.88^a^	7.51 ± 0.18^i^	26.65 ± 0.16^i^
**T**_**1**_	42.21 ± 0.65^a^	5.27 ± 0.08^ab^	13.15 ± 0.49^gh^	56.06 ± 0.67^ab^	7.75 ± 0.08^fghi^	27.35 ± 0.40^hi^
**T**_**2**_	40.74 ± 0.32^ab^	5.13 ± 0.05^abc^	14.58 ± 0.55^efg^	53.74 ± 0.72 cd	8.08 ± 0.11^cdef^	29.70 ± 0.17^efg^
**T**_**3**_	38.57 ± 0.11 cd	4.78 ± 0.04^de^	16.38 ± 0.39 cd	49.16 ± 0.87^fg^	8.41 ± 0.16^bc^	32.33 ± 0.46^c^
**T**_**4**_	36.71 ± 0.61^efg^	4.38 ± 0.07^gh^	18.53 ± 0.18^b^	45.18 ± 0.53^h^	9.05 ± 0.11^a^	36.10 ± 0.56^a^
**T**_**5**_	41.49 ± 0.23^a^	5.16 ± 0.08^abc^	13.30 ± 0.37^gh^	55.91 ± 0.56^ab^	7.87 ± 0.08^efgh^	27.87 ± 0.23^hi^
**T**_**6**_	39.34 ± 0.31^bc^	4.97 ± 0.04 cd	15.11 ± 0.52^def^	53.71 ± 0.72 cd	8.11 ± 0.16^cde^	30.76 ± 0.35^de^
**T**_**7**_	37.83 ± 0.81^cdef^	4.55 ± 0.07^efg^	17.70 ± 0.74^bc^	47.75 ± 0.47^g^	8.58 ± 0.14^b^	33.76 ± 0.85^b^
**T**_**8**_	34.08 ± 0.78^h^	4.05 ± 0.09^i^	20.71 ± 0.88^a^	41.82 ± 0.89^i^	9.19 ± 0.12^a^	36.84 ± 0.47^a^
**T**_**9**_	41.31 ± 0.23^a^	5.19 ± 0.07^abc^	13.53 ± 0.37^fgh^	57.12 ± 0.82^ab^	7.59 ± 0.08^hi^	26.94 ± 0.25^i^
**T**_**10**_	39.17 ± 0.59^bc^	5.02 ± 0.13^bcd^	15.78 ± 0.54^de^	55.74 ± 0.61^b^	7.72 ± 0.08^ghi^	27.72 ± 0.44^hi^
**T**_**11**_	37.18 ± 45^def^	4.80 ± 0.04^de^	17.78 ± 0.70^bc^	52.09 ± 0.57^de^	7.89 ± 0.07^efgh^	29.29 ± 0.15^fg^
**T**_**12**_	35.16 ± 0.65^gh^	4.46 ± 0.11^fgh^	20.86 ± 0.76^a^	48.84 ± 0.47^g^	8.12 ± 0.07^cde^	31.12 ± 0.32 cd
**T**_**13**_	40.87 ± 0.48^ab^	5.14 ± 0.07^abc^	13.46 ± 0.18^fgh^	56.68 ± 0.30^ab^	7.65 ± 0.13^hi^	27.04 ± 0.27^i^
**T**_**14**_	38.30 ± 0.63^cde^	4.94 ± 0.06 cd	15.80 ± 0.59^de^	55.15 ± 0.63^bc^	7.83 ± 0.07^efghi^	28.72 ± 0.87^gh^
**T**_**15**_	36.15 ± 0.74^fg^	4.64 ± 0.11^ef^	18.15 ± 0.74^b^	51.02 ± 0.55^ef^	8.04 ± 0.09^defg^	30.22 ± 0.37^def^
**T**_**16**_	33.85 ± 0.38^h^	4.21 ± 0.07^hi^	21.41 ± 0.62^a^	47.30 ± 0.25^g^	8.25 ± 0.08^bcd^	32.34 ± 0.55^c^
**F value**	70.5**	61.9**	80**	157**	52.1**	141**

Values are represented as Mean±SD. Means bearing different superscript varied significantly

(P<0.01) ^**^ = Highly Significant

The hardness of various cookies sample showed that it fluctuated from 12.26 to 21.41 N, which was enhanced by elevating the amount maize flour addition into the wheat flour. Control having 100% wheat flour had maximum thickness and lowest thickness was noticed in T_16_. The addition of maize hybrids shown greater hardness of composite cookies due to their hard or flinty endosperm in comparison to the maize varieties supplemented cookies. The higher protein and amylose contents in hybrids may slightly intensify the firmness which leads to compact dough and finally lowered moisture retention [56,57]. Maize flour tends to absorb moisture differently and slowly as compared to the wheat flour due to comparatively low amount of gluten, which resulted in drier dough and their cookies to be more hard [58,59]. A similar investigation on composite flour cookies containing wheat, oats, amaranth and sorghum was correlated with our research. The firmness enhanced as the amount of non-wheat sources supplementation increased. In the control (having only wheat) cookies, firmness was detected 30.6 N, after that progressively increased (48.28 N) in 25% substitution [60].

Moreover, the brightness of different cookies samples revealed that its value was diminished by rising the amount of maize flour. L value of cookies ranged from 41.82 to 57.73, maximum brightness was detected in control whereas lowest level was revealed by T_8_. The color a* value or redness was found highest in T_8_ and T_4_ while minimum redness value was displayed by control or T_0_. The value of a or redness was enhanced by rising the amount of maize flour particularly yellow maize flour. Likewise, color b* value or yellowness was augmented with the addition of maize flour substitution into the wheat flour. The range of yellowness fluctuated from 26.65–36.84, which was highest in T_8_ followed by T_4_ and least value of yellowness was presented by control containing 100% maize flour. The color characteristics of cookies revealed a decrease in brightness which was higher in yellow maize opposed to the white maize due to the carotenoids in the yellow cultivars [61]. However, redness and yellowness were increased in cookies as the amount of maize increased and it was higher in yellow contrasted with the white maize incorporated cookies.

The outcomes of color characteristics was in agreement with previous findings for the development of composite flour cookies. The composite blends cookies had darker color to some extent as compared to control or wheat flour cookies [62,63]. In recent research carried out on composite flour cookies containing wheat and hulless barley flour revealed similar outcomes. There was seen a momentous reduction in the L value from 66.18 (100% wheat flour cookies) to 62.90 for (100% barley flour cookies) and a substantial rise in the redness or a* value from 5.49 (100% wheat flour cookies) to 7.48 (100% barley flour cookies) that was in accordance with our findings. Whereas a reduction in the yellowness/b* value was observed from 36.51 (100% wheat flour) to 31.68 (100% barley flour cookies) which showed deviation from our findings [64].

### Sensory evaluation of cookies

**Fig 2:**
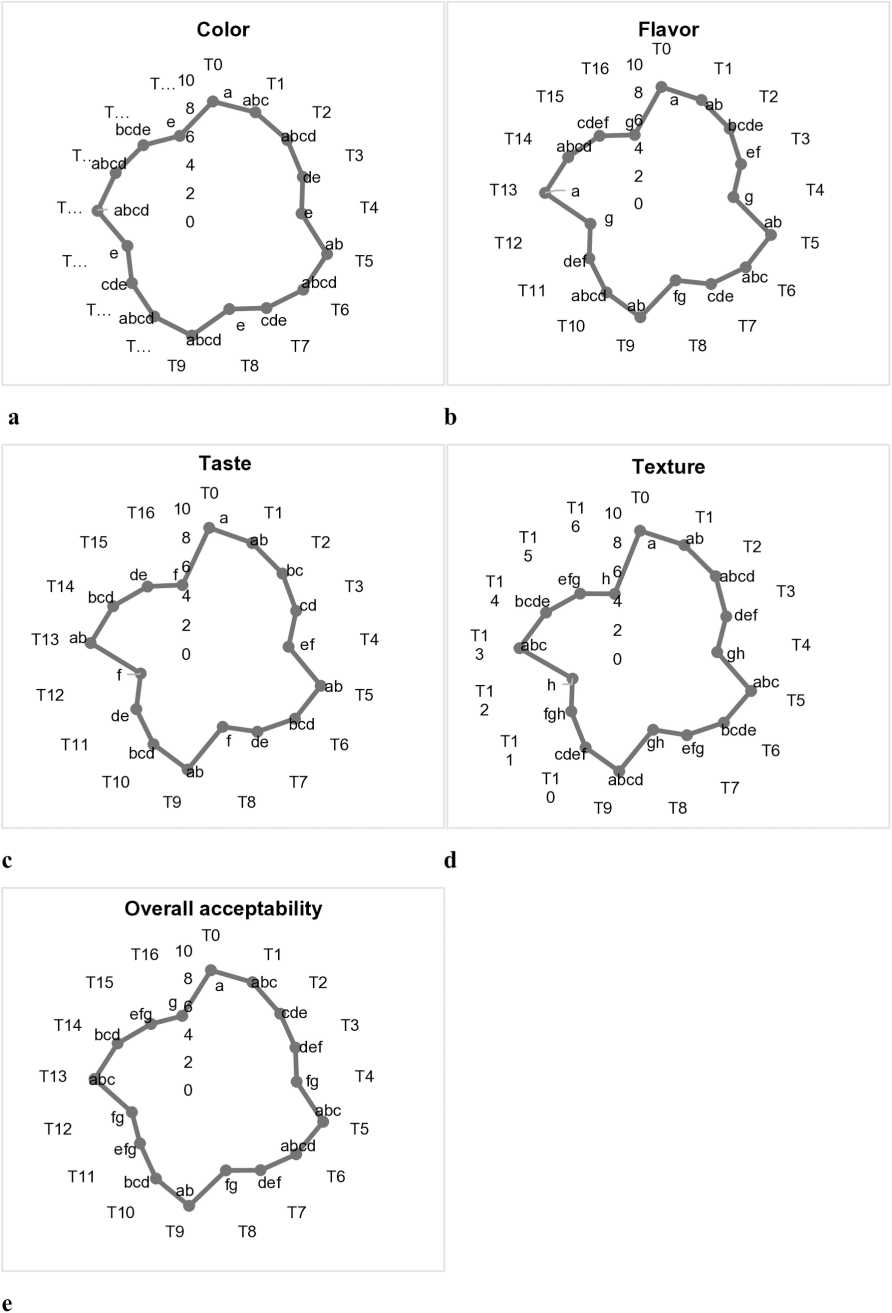
Sensory evaluation of cookies; color **(a)**, flavor (b) taste **(c)**, texture (d) and overall acceptability **(e)**. Means bearing different superscript varied significantly.

A highly significant variation was observed among the different characteristics, e.g., color, flavor, taste, *etc*. of sensory evaluation as displayed in Fig 2. The color score values of cookies samples were varied from 6.3–8.5 which was found lowest in treatments T_4_, T_8_ and T_12_, while peak score was attained in control comprising 100% common flour. Likewise, the flavor score was found highest in control and T_13_ (8.4), while minimum score was seen in T_4_ (5.2). The addition of maize flour negatively impacted the sensorial evaluation of cookies, and its score was decreased with the substitution of maize flour into the wheat flour. Additionally, taste was found to diminish with level of maize flour substitution in the wheat flour and maximum score was observed in control (8.7) whereas least score was attained in T_12_ preceded by T_8_ and T_16_. The score values of texture fluctuated from 4.8 to 8.8, maximum was attained by control (100% wheat flour) and least score was shown by T_12_ and T_16_. The overall acceptability of cookies samples was decreased by enhancing the amount of maize flour substitution into the common flour. The greatest score for overall acceptability was observed in control (8.6) subsequent by T_9_, T_5_, T_13_, T_1_, whereas least score was detected in T_16_.

The sensory evaluation of composite cookies showed that yellow maize incorporated cookies has better consumer acceptance as compared to the white due to their yellowish color, distinct flavor and texture. Likewise, composite cookies made with the yellow varieties or hybrids of maize were mostly ranked higher for their color, flavor and overall acceptability due to pleasing golden hue and gentle toasty aroma. The decline in sensory might be attributed to different factors inherent in the maize flour, contributing a characteristic texture and flavor, lead to a decrease in smoothness and uniformity. Wheat flour contains gluten that gives structure and assists with the soft, chewy texture of wheat cookies. Whereas, it is less or deficient in all other non wheat sources comparatively, which resulted in lower or poor sensory performance [65, 66, 67].

Our findings were correlated with the study of the wheat maize composite cookies supplemented with walnut seed protein isolates. The outcomes for the sensory characteristics declined as the amount of wheat flour was decreased into the composite blends. The overall acceptability was reduced from 9.0 to 4.27 by decreasing the amount of wheat flour from 100 to 50%. Similar diminishing scores of sensory were attained in other parameters such as taste, appearance, crispiness, mouthfeel, and aroma [68]. A study conducted on wheat maize composite blends cookies revealed the analogous outcomes for the sensory characteristics. The mean score values decreased with the increasing amount of maize flour from 0 to 100% into the wheat flour [69]. Comparable sensorial behavior was found in buckwheat cookies supplemented wheat flour. The scores of organoleptic properties revealed that gradual increase of wheat flour into the cookies enhanced the consumer acceptance [70].

## Conclusion

Incorporation of maize into the wheat flour for the development composite blends and subsequent product enhanced the nutritional profile such as increase in crude fat, fiber and ash contents while reduction in phytates. The pasting behavior as analyzed by rapid visco analyzer showed an inverse relation with the maize flour addition on their viscosities. The physical attributes of cookies showed an increase in hardness, redness, and yellowness while decrease in diameter, thickness and brightness by increasing the amount of maize flour replacement into the wheat flour. It was concluded from the outcomes that supplementation of maize into wheat flour gradually affected the consumer acceptance as well as the physical properties of cookies and found acceptable up to 30% substitution. Therefore, appropriate efforts should be made to counter these unfavourable effects. The gradual decrease in physical and sensory attributes of wheat maize composite cookies could be enhanced by adding the lecithin, glycerol and xanthan gum that improves the moisture retention, dough viscosity and texture attributes. Moreover, whey protein, milk solids and caramel could be added to enhance the color and flavor characteristics of the composite cookies. Commercially, it will lower the burden on the wheat as well as its import. Moreover, products other than the cookies need to be formulated in order to add maize flour to our nourishment. The composite flours by using the wheat along with non-wheat sources, e.g., maize, chickpea, barley, *etc.* need to be encourage for diversifying our diet. Moreover, desirable technologies/treatments need to be planned in order to improve the utilization of non-wheat sources, so that these non wheat cereals can be used at higher levels into the wheat flour for the development of bakery products.

## Supporting information

S1 Protocol Proximate composition. (PDF)

S1 Table Phytic acid (mg/100g) of wheat flour and composite blends. Values are represented as mean±SD. Means bearing different superscript varied significantly.
(PDF)

S2 Table Sensory evaluation of cookies. Values are represented as Mean±SD. Means bearing different superscript varied significantly.
(PDF)
