# Supplementary material for: Development of cookies from wheat-yellow/white maize composite blends and their physical and sensory evaluation
Source: PLoS One. 2025 Jun 18;20(6):e0326532. doi: 10.1371/journal.pone.0326532 (PMC12176214; doi:10.1371/journal.pone.0326532)
Supplement: S1 Protocol — (PDF) [file pone.0326532.s001.pdf]

Proximate composition

Common and composite flours were examined for their proximate composition that includes crude proteins, fats, ash contents, crude fibers, moisture contents, and nitrogen free extract (NFE) following the procedures given in AACC (2010).

Moisture

The moisture contents were checked by using the method no. 44-15.02. as mentioned in the AACC (2010). Samples were minced thoroughly and brought in the china dishes that were weighed up earlier and then dried out using the hot-air oven at the temperature of 105±5 ^{o}C until weight became constant. After the drying process, these samples were detached and placed in the desiccator (approximately 5-10 minutes). The purpose of placing these hot samples into the desiccator was to cool them down without absorption of atmospheric moisture. After that, trials were weighed, and their moisture contents were calculated by using the resulting formula.

$$\text{Moisture (\%)} = \frac{\text{trial's mass before drying (g)} - \text{trial's mass after drying (g)}}{\text{trial's mass before drying (g)}} \times 100$$

Crude protein

The crude protein of the replicas was measured by Kjeldhal apparatus as mentioned in AACC (2010) method no. 46-13.01.

Digestion

About 0.5-2.0 g of sample for protein analysis was collected in the digestion chamber. Further, 5 g of digestion tablet/mixture were added in it and 30 mL of concentrated sulphuric acid was also transferred. For the breakdown process, the combination was left for almost 3-4 hours or until transparent or bright green color detected in digestion

chamber. Subsequently, it was cooled for 30 minutes. Afterward, it was transported to a volumetric flask and prepared amount up to 250 mL by using distilled water.

Distillation assembly

Distillation was performed by using 10 mL of previously processed sample and 10 mL sodium hydroxide (40%) in its chamber. Accordingly, ammonia fumes were liberated and entrapped in borate solution (4%) comprising 2-3 droplets of methyl red indicator. As a result, borate and methyl red solution changed from red to light-yellow color.

Titration

Later, obtained distillate was titrated against 0.1 N sulphuric acid (H_2SO_4) solution until light/pink color appeared. The crude proteins were determined by multiplying obtained nitrogen content with a specific factor (6.25)

$$\text{Nitrogen \%} = \frac{\text{Vol. of 0.1 N sulphuric acid} \times \text{Dilution Vol. (250 mL)} \times .0014}{\text{Dilution Vol. taken} \times \text{Sample weight}} \times 100$$

Crude protein = N (%) × 6.25

Crude fiber

The analysis of fiber contents was done according to the procedure outlined in AACC (2010) method no. 32-10.01. At first, we prepared the lipid free sample by using the soxhlet apparatus following the method as mentioned below in crude fat analysis. In this regard, about 2 g of water and lipid-free samples were taken, and digestion was done in 1.25% H_2SO_4 for 30 minutes followed by digestion in 1.25% NaOH for 30 minutes. After treating with H_2SO_4 and NaOH, material was washed and then filtered by using Wattsman filter paper. The remaining residue was dried in a hot air oven till all moisture was removed. Then, the samples were weighed and placed for 4-5 hours

at temperature of 550-650 °C in the muffle furnace until white or grey color appeared. To calculate crude fiber the following formula was used;

$$\text{Crude fiber (\%)} = \frac{\text{Mass loss}}{\text{Wt. of the sample (g)}} \times 100$$

Crude fat

Soxhlet apparatus was employed to measure the crude fats of experiments using the technique as mentioned in AACC (2010) method no. 30-25.01. For fat contents determination, 5g moisture free samples were gathered in separate thimbles which were made of filter papers and thimbles were kept in an extraction chamber of Soxhlet. The heater temperature was accustomed in a way that the ether droplets fell continuously on the trial placed in the chamber. For the procedure of fat extraction, hexane was used. Fat content was obtained in 4 hours approximately when 6-7 siphons completed. After that, the samples were located in the oven for 2-3 hours to evaporate solvent. Then, these samples were placed in desiccator to avoid moisture absorption from atmosphere and to cool them down. Samples were weighed and value of fat percentage was obtained by using the below mentioned formula.

$$\text{Crude fat (\%)} = \frac{\text{Weight of trial before washing} - \text{Weight of trial after washing}}{\text{Weight of trial before washing}} \times 100$$

Ash contents

Composite flours and wheat flour were examined for ash content by employing the procedure discussed in AACC (2010) procedure no. 08-01.01. Well ground solid samples (5-10 g) were placed in the crucibles which were weighed earlier. Afterward, charring was performed using the burner. Then, samples were placed in muffle

furnace and temperature 550-650 ^{o}C was managed till 4-5 hours that changed the samples into greyish white color residues. Finally, place it in the desiccator to cool it down, without gaining any moisture from the environment.

Calculation was done according to the below mentioned formula

$$\text{Ash (\%)} = \frac{\text{Mass of residues}}{\text{Wt. of the sample}} \times 100$$

Nitrogen free extract

NFE was measured using the below mentioned formula

NFE = 100- (M % + CF % + CP % + CF % + Ash %)

M = Moisture

CF = Crude fat

CP = Crude protein

CF = Crude fiber
