# Supplementary material for: Development of cookies from wheat-yellow/white maize composite blends and their physical and sensory evaluation
Source: PLoS One. 2025 Jun 18;20(6):e0326532. doi: 10.1371/journal.pone.0326532 (PMC12176214; doi:10.1371/journal.pone.0326532)
Supplement: S1 Table — Values are represented as mean±SD. Means bearing different superscript varied significantly. (PDF) [file pone.0326532.s002.pdf]

S1 Table. Phytic acid (mg/100g) of wheat flour and composite blends. Values are represented as mean±SD. Means bearing different superscript varied significantly.

Treatment	Phytic acid
T_0	314.22 ± 10.27^{a}
T_1	288.48 ± 13.65^{ab}
T_2	272.17 ± 7.60^{bcd}
T_3	257.27 ± 14.20^{bcdef}
T_4	213.08 ± 9.03^{hi}
T_5	281.35 ± 12.77^{b}
T_6	268.97 ± 13.68^{bcde}
T_7	248.61 ± 7.41^{cdefg}
T_8	210.89 ± 6.34^{i}
T_9	277.65 ± 11.64^{bc}
T_{10}	243.87 ± 5.98^{defgh}
T_{11}	228.22 ± 13.58^{fghi}
T_{12}	207.80 ± 9.96^{i}
T_{13}	279.31 ± 12.94^{bc}
T_{14}	266.84 ± 7.24^{bcde}
T_{15}	238.74 ± 11.96^{efghi}
T_{16}	218.76 ± 5.97^{ghi}
