# Supplementary material for: Development of cookies from wheat-yellow/white maize composite blends and their physical and sensory evaluation
Source: PLoS One. 2025 Jun 18;20(6):e0326532. doi: 10.1371/journal.pone.0326532 (PMC12176214; doi:10.1371/journal.pone.0326532)
Supplement: S2 Table — Values are represented as Mean±SD. Means bearing different superscript varied significantly. (PDF) [file pone.0326532.s003.pdf]

S2 Table. Sensory evaluation of cookies. Values are represented as Mean±SD. Means bearing different superscript varied significantly.

Treatment	Color	Flavor	Taste	Texture	Overall acceptability
**T_0**	$8.5±0.70^{a}$	$8.4±0.84^{a}$	$8.7±0.67^{a}$	$8.8±0.42^{a}$	$8.6±0.51^{a}$
**T_1**	$8.3±0.67^{abc}$	$8.0±0.47^{ab}$	$8.2±0.42^{ab}$	$8.4±0.51^{ab}$	$8.3±0.48^{abc}$
**T_2**	$7.8±0.78^{abcd}$	$7.3±0.67^{bcde}$	$7.5±0.70^{bc}$	$7.7±0.48^{abcd}$	$7.4±0.69^{cde}$
**T_3**	$7.1±0.31^{de}$	$6.4±0.69^{ef}$	$6.7±0.82^{cd}$	$6.6±0.69^{def}$	$6.8±0.63^{def}$
**T_4**	$6.3±0.67^{e}$	$5.2±0.63^{g}$	$5.5±0.84^{ef}$	$5.3±0.94^{gh}$	$6.2±0.63^{fg}$
**T_5**	$8.4±0.51^{ab}$	$8.2±0.63^{ab}$	$8.0±0.81^{ab}$	$7.9±0.73^{abc}$	$8.4±0.69^{abc}$
**T_6**	$8.0±0.94^{abcd}$	$7.6±0.84^{abc}$	$7.4±0.69^{bcd}$	$7.2±0.63^{bcde}$	$7.7±0.82^{abcd}$
**T_7**	$7.2±0.63^{cde}$	$6.8±0.78^{cde}$	$6.3±0.82^{de}$	$6.1±0.99^{efg}$	$6.8±0.78^{def}$
**T_8**	$6.3±1.15^{e}$	$5.6±0.84^{fg}$	$5.1±0.87^{f}$	$4.9±0.87^{gh}$	$5.9±0.87^{fg}$
**T_9**	$8.2±0.63^{abcd}$	$8.3±0.48^{ab}$	$8.1±0.73^{ab}$	$7.8±0.63^{abcd}$	$8.5±0.70^{ab}$
**T_{10}**	$7.9±0.87^{abcd}$	$7.5±0.52^{abcd}$	$7.3±0.82^{bcd}$	$7.1±1.10^{cdef}$	$7.5±0.70^{bcd}$
**T_{11}**	$7.2±0.63^{cde}$	$6.5±0.52^{def}$	$6.3±0.82^{de}$	$5.9±0.87^{fgh}$	$6.4±0.84^{efg}$
**T_{12}**	$6.3±0.67^{e}$	$5.3±0.67^{g}$	$4.9±0.73^{f}$	$4.8±0.63^{h}$	$5.9±0.56^{fg}$
**T_{13}**	$8.2±0.63^{abcd}$	$8.4±0.51^{a}$	$8.2±0.42^{ab}$	$8.3±0.67^{abc}$	$8.4±0.51^{abc}$
**T_{14}**	$7.7±0.67^{abcd}$	$7.5±0.84^{abcd}$	$7.4±0.69^{bcd}$	$7.2±0.63^{bcde}$	$7.5±0.52^{bcd}$
**T_{15}**	$7.3±0.48^{bcde}$	$6.6±0.51^{cdef}$	$6.3±0.82^{de}$	$6.1±0.99^{efg}$	$6.4±0.69^{efg}$
**T_{16}**	$6.5±0.70^{e}$	$5.3±0.94^{g}$	$5.1±0.73^{f}$	$4.8±0.78^{h}$	$5.7±0.82^{g}$
